# The impact of posture changes on critical closing pressure and resistance-area product regulation in healthy subjects

**DOI:** 10.1007/s00421-025-05972-2

**Published:** 2025-09-22

**Authors:** X. Zhong, R. H. Clough, R. B. Panerai, J. S. Minhas

**Affiliations:** 1 Cerebral Haemodynamics in Ageing and Stroke Medicine (CHiASM) Research Group, Department of Cardiovascular Sciences, University of Leicester, Leicester, UK; 2 NIHR Leicester Biomedical Research Centre, British Heart Foundation Cardiovascular Research Centre Excellence, Glenfield Hospital, Leicester, UK

**Keywords:** Cerebral blood flow, Posture, Vasomotor reactivity

## Abstract

**Introduction:**

Cerebral blood flow (CBF) is affected by posture changes, but there is a paucity of research examining the effect of posture on dynamic cerebral autoregulation (dCA). The step responses of critical closing pressure (CrCP) and resistance area product (RAP) obtained from the cerebral blood velocity (CBv) signal can reflect the changes in dCA, enabling exploration of dCA changes in supine, sitting and upright postures.

**Methods:**

In 22 participants (11 males, aged 30.2 ± 14.3 years), two recordings were made for each posture, corresponding to supine, sitting, and standing. Blood pressure (BP, Finometer), MCAv and PCAv (transcranial Doppler ultrasound), end-tidal carbon dioxide (EtCO_2_, capnography) and heart rate (ECG) were continuously recorded. CrCP and RAP were obtained for each cardiac cycle, and the step responses of CBv (SRV_MCA/PCA_), CrCP (SRV_CrCP_), and RAP (SRV_RAP_), for both arteries, were calculated after subcomponent and transfer function analysis.

**Results:**

Moving from supine to sitting, and then standing, led to reductions in mean MCAv (*p* < 0.001), PCAv (*p* = 0.037), BP (*p* < 0.001) and EtCO_2_ (*p* < 0.001), accompanied by changes in SRV_MCA_ (*p* = 0.002), but not in SRV_PCA_ (*p* = 0.78). For both arteries, SRV_RAP_ and SRV_CrCP_ reflected changes in posture (both *p* < 0.001).

**Conclusion:**

Posture changes can significantly affect the step responses of MCAv, CrCP, and RAP. The interaction between posture and EtCO_2_ from the perspective of the CrCP and RAP step responses needs further exploration in future studies.

## Introduction

Standing, from the sitting or supine position, leads to changes in multiple physiological variables, such as arterial blood pressure (BP), cardiac output (CO), heart rate (HR), stroke volume (SV), lung ventilation, and in the distribution of blood volumes, mediated by autonomic nervous system regulatory mechanisms (Khan et al. 2025; Schondorf 2023). In patients with orthostatic intolerance, there is inadequate adjustment in cerebral blood flow (CBF) in the standing position, leading to symptoms such as dizziness, fatigue, brain fog, and fainting (Khan et al. 2025; Novak 2016; Sheldon et al. 2015). Under normal physiological conditions, the mechanisms of cerebral autoregulation (CA), in response to changes in BP, and vasomotor reactivity (VMR), in response to changes in PaCO_2_ (Immink et al. 2006), tend to maintain CBF within narrow limits, to prevent the occurrence of episodes of hypo- or hyper-perfusion (Willie et al. 2014). However, the behaviour of these mechanisms, in response to changes in posture, is still poorly understood (Clough et al. 2024; Garrett et al. 2017; Gelinas et al. 2012; Gisolf et al. 2004; Serrador et al. 2006; Shoemaker et al. 2001). In a relatively large number of studies, the dominant view has been that dynamic CA was not compromised in orthostatic intolerance (Mankoo et al. 2023). Nevertheless, it is possible that this conclusion has been the result of methodological limitations of the techniques adopted for dCA assessment (Castro et al. 2017; Kostoglou et al. 2024; Panerai et al. 2023). As a first step towards addressing this potential limitation, we have attempted to provide a more sensitive description of the changes that take place in dCA, due to changes in posture in a healthy population, by using a more advanced approach, involving the dynamic behaviour of critical closing pressure (CrCP) and resistance-area product (RAP). CrCP is the BP value at which CBF becomes zero, due to an insufficient transmural pressure to counteract the active tension of the vessel wall (Burton 1951; Panerai 2003). The RAP is the inverse slope of the instantaneous cerebral blood velocity(CBv)-ABP relationship, as measured with transcranial Doppler, considering that CBF is the product between CBv and vessel cross-sectional area (Evans et al. 1988). Changes in CrCP and RAP, in response to changes in posture, have been reported (Carey et al. 2001; Robertson et al. 2014), but recent advances in cerebrovascular modelling have shown that the mean arterial pressure (MAP)-CrCP and MAP-RAP transfer functions have the potential to provide additional, more sensitive information, to reflect physiological changes, and hence hold considerable promise to express myogenic and metabolic changes that can take place during changes in posture (Panerai et al. 2021, 2020; Schondorf 2023; Willie et al. 2014). In summary, we tested the hypothesis that the CrCP and RAP responses to changes in MAP can reflect adjustments in cerebral haemodynamics that take place during changes in posture.

## Methods

### Experimental procedures

This retrospective analysis investigated data collected from healthy participant studies at the University of Leicester (Clough et al. 2024).

The protocol consisted of six recordings, each 10 min in duration. Two recordings were taken when the participant was supine, two were taken when the participant was sitting, and two were taken when the participant was standing up. The original recordings consisted of three minutes of breathing at rest, three minutes of modulated CO_2_ ventilation, then a further two minutes of breathing at rest. Therefore, there was a washout period between every recording. Only the poikilocapnic breathing at the beginning of each recording was used in the present study (Clough et al. 2024).

### Physiological measurements

Staff and students from the University of Leicester, aged 18 years or older were enrolled, excluding pregnant or lactating women and those with major cardiovascular, neurological, or respiratory disease. Those with properly controlled hypertension on a single antihypertensive agent were also included. Participants did not consume heavy meals, alcohol, caffeine, cigarette smoking, for four hours or performed strenuous exercise for 12 h prior to the study. The phase of the menstrual cycle of female participants was noted (Clough et al. 2024). Trials were conducted in the temperature-regulated (20–24 °C) quiet environment of the Cerebral Haemodynamics in Ageing and Stroke Medicine (CHiASM) Research Group Laboratory, University of Leicester. Transcranial Doppler-measured CBv in the dominant middle cerebral artery (MCAv) and non-dominant posterior cerebral artery (PCAv) were utilised (Viasys Companion III; Viasys Healthcare, Beckton Dickinson & Co, Franklin Lakes, NJ). Real-time monitoring included end-tidal CO₂ (EtCO_2_, nasal prongs, Salter laboratories) with infrared capnography (Capnocheck Plus; Smiths Medical, Ashford, UK)), heart rate (three-lead ECG), and BP continuous artery volume clamping of the digital artery (Finometer, Finapres Medical Systems, Amsterdam, The Netherlands). The Physiocal servo-correction algorithm was switched off before each recording. Systolic and diastolic brachial blood pressure were measured before each recording with sphygmomanometry (OMRON Model 705IT; Omron Corp., Kyoto, Japan). All variables were recorded at 500 samples/s using the PHYSIDAS data acquisition system (Department of Medical Physics, University Hospitals of Leicester, Leicester, UK).

### Data analysis

All signals underwent visual removal of artefacts and noise, and narrow spikes (< 100 ms) were removed using linear interpolation. CBv channels were median-filtered, and all signals were filtered with an eighth-order Butterworth low-pass filter (cutoff 20 Hz). BP was calibrated against brachial systolic/diastolic measurements at recording initiation. R-R intervals were automatically derived from ECG for the computation of beat-to-beat HR, and manually corrected for missing intervals (Panerai et al. 2021, 2020). Mean, systolic, and diastolic BP and CBv were calculated per cardiac cycle, and CrCP and RAP were estimated based on the first harmonic method (Panerai et al. 2011). End-points of the expiratory phase of EtCO₂-derived data were automatically detected, linearly interpolated, and synchronised to the cardiac cycle. All beat-to-beat parameters were spline interpolated and resampled at 5 samples/s to generate a uniform timebase. Hypercapnic/hypocapnic post-episode data were excluded, with two ~ 200 s segments of baseline data remaining to analyse for each subject, at each posture.

TFA of the MAP-MCAv and MAP-PCAv relationships used Welch's method with Hanning windowing to reduce spectral leakage (Claassen et al. 2016; Welch 1967). Coherence, gain, phase, and step responses were derived from smoothed auto-/cross-spectra, averaged from six 102.4 s segments of data with 50% superposition, extracted from the two recordings available for each posture. (Claassen et al. 2016; Panerai et al. 1998). Dynamic CA was quantified by the ARI, based on fitting CBv step responses to Tiecks' model curves (normalized mean squared error, NMSE ≤ 0.30; 0.15–0.25 Hz coherence > 95% confidence limit) (Panerai et al. 2016; Tiecks et al. 1995).

### Subcomponent analysis

Assuming linearisation of the instantaneous BP-CBv relationship, for each cardiac cycle CBv can be expressed as CBv = (BP − CrCP)/RAP (Panerai 2003). For small changes in RAP, the corresponding change in CBv can be expressed as the sum of the changes in MAP, CrCP and RAP for each cardiac cycle (Panerai et al. 2005). Subcomponent analysis corresponds to the normalisation of all these changes, leading to:

1 $${\mathrm{V}}_{{{\mathrm{CBv}}}} \, = \,{\mathrm{V}}_{{{\mathrm{MAP}}}} \, + \,{\mathrm{V}}_{{{\mathrm{CrCP}}}} \, + \,{\mathrm{V}}_{{{\mathrm{RAP}}}}$$

Where the terms on the right side of equation [1] correspond to the subcomponents, expressing the percentage contribution of MAP, CrCP, and RAP to changes in CBv (Panerai et al. 2020, 2005). Mean values for CBv, MAP, CrCP, and RAP over 5-min intervals were calculated for normalization (Table 1), leading to beat-to-beat deviations (Δv, Δp, Δc, Δr) for CBv (MCAv or PCAv), MAP, CrCP and RAP, respectively.

**Table 1:** Main physiological parameters during supine, sitting, and standing positions

Parameter	Supine (*n* = 22)	Sit (*n* = 22)	Stand (*n* = 22)	*p*-value
PCAv (cm/s)	41.9 (15.2)	42.4 (14.8)	38.8 (13.5)	**0.037**
MCAv (cm/s)	61.9 (20.9)	57.6 (18.6)	54.4 (17.2)	** < 0.001**
MAP (mmHg)	83.5 (12.8)	66.4 (9.6)	70.0 (12.9)	** < 0.001**
Heart rate (beats/min)	69.6 (11.3)	69.6 (9.8)	83.2 (14.3)	** < 0.001**
EtCO_2_ (mmHg)	37.1 (2.5)	36.3 (3.0)	34.1 (3.2)	** < 0.001**
PCA-CrCP (mmHg)	21.0 (12.7)	12.0 (13.6)	14.5 (14.4)	** < 0.001**
PCA-RAP (mmHg.s/cm)	1.83 (0.90)	1.51 (0.79)	1.77 (0.91)	0.058
MCA-CrCP (mmHg)	22.7 (12.6)	14.6 (12.2)	19.8 (11.3)	**0.003**
MCA-RAP (mmHg.s/cm)	1.25 (0.97)	1.02 (0.43)	1.08 (0.40)	0.26

Values are mean (SD) with corresponding values of *n*

*PCAv* Posterior artery blood velocity, *MCAv* Middle cerebral artery blood velocity, *MAP* Mean arterial blood pressure, *EtCO*_*2*_ End-tidal CO_2_, *CrCP* Critical closing pressure, *RAP* Resistance-area product

*p* values from one-way repeated-measures ANOVA. Values in bold correspond to *p* < 0.05

Based on linear Fourier transform behavior, a step change in MAP leads to corresponding changes in the other variables that equate to their step responses with similar terms to Equation [1]. By employing algebraic rearrangement of terms, the MAP step change can then be written as (Panerai et al. 2020):

2 $${\mathrm{SRV}}_{{{\mathrm{MAP}}}}^{*} \, = \,{\mathrm{SRV}}_{{{\mathrm{CBv}}}} \, - \,{\mathrm{SRV}}_{{{\mathrm{CrCP}}}} \, - \,{\mathrm{SRV}}_{{{\mathrm{RAP}}}}$$

SRV_MAP_^*^ here refers to the estimate of MAP step response as the arithmetic sum of the three sub-component step responses SRV_CBv_, SRV_CrCP_, and SRV_RAP_. The above equation thus serves as a checksum to assess the internal consistency and integrity of the other three step responses (Panerai et al. 2024, 2021). Equation [2] applies equally to CBv values obtained from the MCA or PCA, with corresponding values for MCA-SRV_CrCP_, PCA-SRV_CrCP_, MCA-SRV_RAP_ and PCA-SRV_RAP_.

Statistical acceptance of SRV_PCA_ and SRV_MCA_ required coherence > 95% confidence limit (0.15–0.25 Hz) and normalized mean squared error (NMSE) ≤ 0.30 for Tiecks model fitting (Panerai et al. 2016; Tiecks et al. 1995). SRV_CrCP_ and SRV_RAP_ were accepted only if their transfer functions exceeded the 90% confidence limit for coherence in at least one hemisphere under different postures. SR values were averaged across three intervals: T1 (0–5 s, peak response), T2 (5–10, plateau onset), and T3 (10–15 s, tail phase).

Mixed-effects repeated ANOVA assessed the effects of time (T1-T3), posture (supine, sitting, standing) and artery (MCA, PCA). For significant effects of the F-test, post-hoc analysis was performed with Tukey’s HSD test. Significance was set at *p* < 0.05.

## Results

A total of 22 subjects (11 males) contributed 132 recordings for analysis. The group was aged 30.2 ± 14.3 years, with a range of 19 to 72 years. There were 16 (72.7%) Caucasian, 6 (27.3%) Asian, and BMI was 21.1 ± 2.7 kg/m^2^. While all were healthy for their age, the following minor exceptions were noted: one had controlled mild hypertension, three were on hormonal contraception (two on combined oral contraceptives, one on progesterone), one smoked two cigarettes per week, and one was an ex-smoker. One protocol deviation was noted in the case of caffeine consumption within 1 h prior to participation.

### Baseline values

**Fig. 1:**
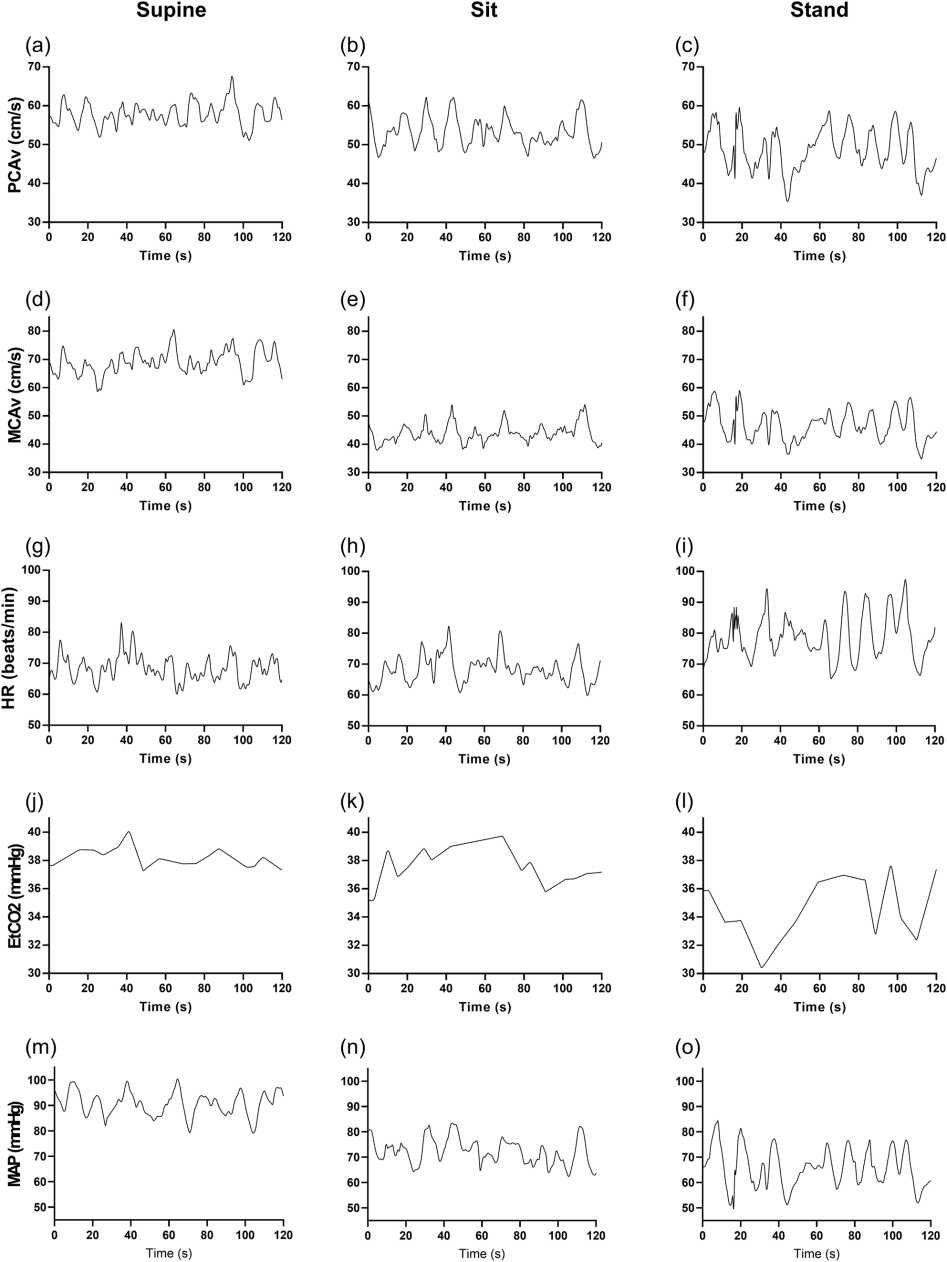
Representative recordings from a 21-yr-old male subject during poikilocapnic breathing in the (**a,d,g,j,m**) supine, (**b,e,h,k,n**) sitting, and (**c,f,i,l,o**) standing position for the (**a,b,c**) posterior cerebral artery blood velocity (PCAv), (**d,e,f**) middle cerebral artery velocity (MCAv), (**g,h,i**) heart rate (HR), (**j,k,l**) end-tidal CO_2_ (EtCO_2_), and (**m,n,o**) mean arterial blood pressure (MAP)

Table 1 compares the differences in baseline data for the 22 participants in the three postures of supine, sitting, and standing. One-way repeated ANOVA shows that mean PCAv (*p* = 0.037) and MCAv (*p* < 0.001) in the standing posture were significantly lower than in the other two postures. There were also significant differences in MAP (*p* < 0.001), with the highest value in the supine position (83.5 ± 12.8 mmHg). Heart rate increased (*p* < 0.001) and EtCO_2_ decreased (*p* < 0.001) in the standing position. For both arteries, CrCP supine was higher than sitting (*p* < 0.025), for the PCA was also higher than standing (*p* = 0.020). These mean value changes are also apparent in the beat-to-beat fluctuations of the main parameters, as represented for a single subject in Fig. 1.

### Step responses – effect of time

**Fig. 2:**
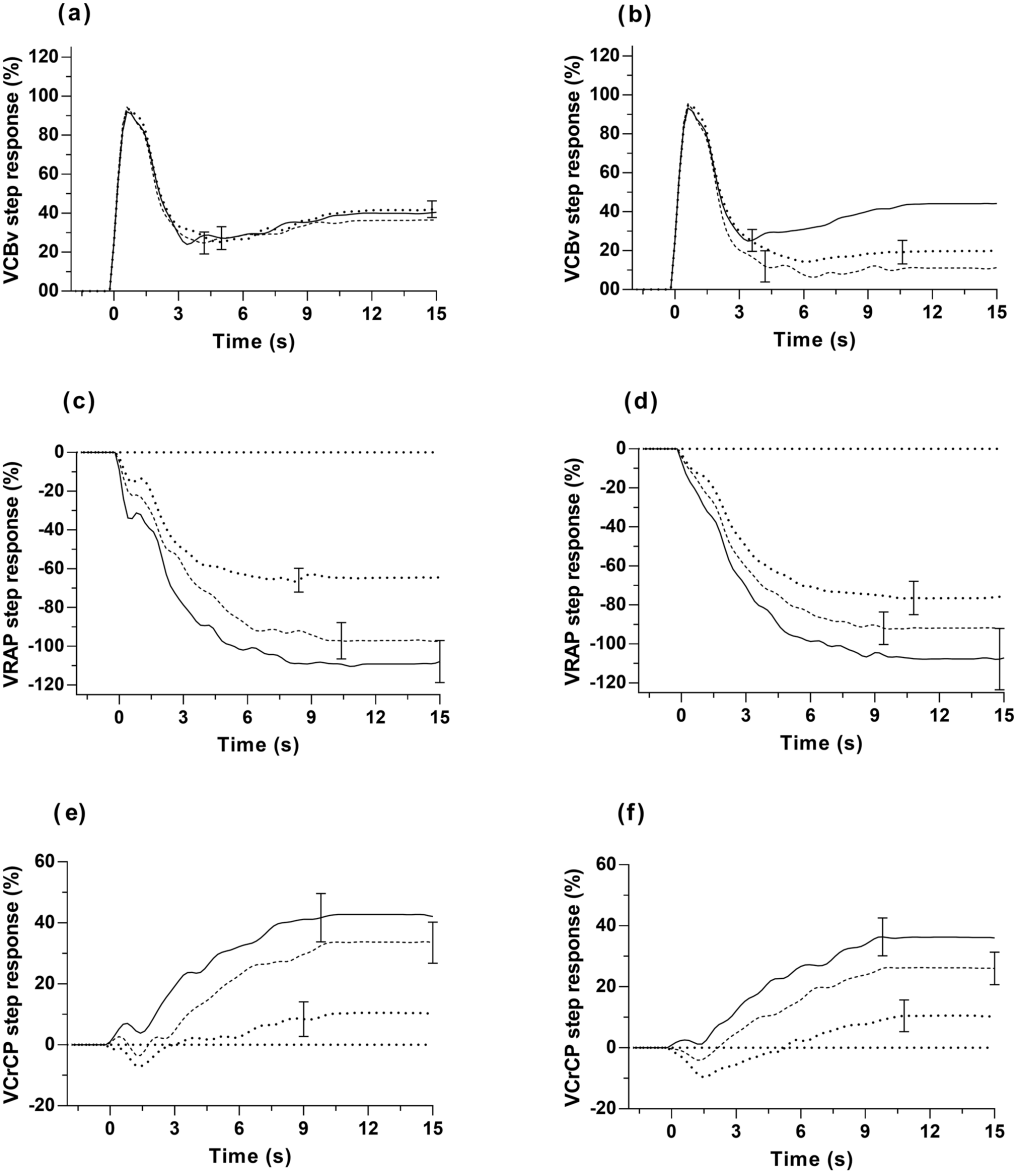
Population average step responses for different postures in supine, sitting and standing. **a** V_PCAv_ step response, **b** V_MCAv_ step response; **c** PCA—V_RAP_ step response, **d** MCA—V_RAP_ step response; **e** PCA—V_CrCP_ step response, **f** MCA—V_CrCP_ step response. The error bars represent largest ± SE at the point of occurrence

One step response of RAP in the supine position, from the PCA, was rejected as an outlier. All step responses showed a strong effect of time with highly significant differences for the time intervals T1-T3 (Fig. 2 and Table 2, all *p* < 0.001). Both SRV_PCAv_ and SRV_MCAv_ increased sharply in response to the MAP upstroke, returning more slowly to the baseline level at the tail (Figs. 2a and b). The RAP step response exhibited a gradual decay pattern and reached stability after approximately 7 s (Figs. 2c and d). The CrCP step responses showed a small oscillation in the time interval 0–2 s, followed by a gradual increase reaching a plateau after approximately 9 s (Figs. 2e and f).

**Table 2:** Repeated measures mixed-effects ANOVA results for the effects of artery (PCA vs MCA), posture (supine, sitting, standing) and time (T1,T2, T3) on sub-component step responses

Step responses	*p*-value artery	*p*-value posture	*p*-value time	Significant interactions
SRV_CBv_	0.003	0.031	< 0.001	artery*posture *p* = **0.001** artery*time *p*** < 0.001** posture*time *p* = **0.001**
SRV_RAP_	0.48	0.002	< 0.001	artery*posture *p* = **0.031** posture*time *p* = **0.031**
SRV_CrCP_	0.16	< 0.001	< 0.001	posture*time = **0.002**

Significant *p*-values for the three main effects and interactions are in bold. See symbols in Tables 3–5 for Tukey’s post-hoc test differences

*SRV*_*CBv*_ step response of the cerebral blood velocity subcomponent for comparison between the posterior cerebral (PCA) and middle cerebral (MCA) arteries, *SRV*_*RAP*_ step response for the resistance-area product subcomponent for comparison between values from the PCA and MCA, *SRV*_*CrCP*_ step response for the critical closing pressure subcomponent for comparison between values from the PCA and MCA

### Step responses – effect of posture

The interaction effects in Table 2, indicated that SRV_PCAv_ was not sensitive to changes in posture (Table 3, Fig. 2a), but SRV_MCAv_ had elevated values at T2 and T3 for the supine position, compared to sitting and standing (post-hoc *p* < 0.001, Table 3, Fig. 2b). For both arteries, SRV_RAP_ showed a gradual decrease in amplitude from supine to sitting to standing, which was highly significant for the PCA-SRV_RAP_ for T2 and T3 (p < 0.001, Table 4, Fig. 2c). For the MCA-SRV_RAP_ though, differences were only significant between supine and standing (Table 4, Fig. 2d).

**Table 3:** Mean step responses for PCAv and MCAv subcomponents for time intervals T1-T3

Artery	Posture	T1	T2	T3
SRV_PCAv_ (%)	supine	48.2 (15.0)^#^	32.3 (23.1)	39.7 (25.4)
	sitting	47.6 (14.2)^#^	30.8 (19.0)	36.1 (20.6)
	standing	49.9 (11.4)^#^	31.9 (16.2)	41.25 (19.6)^*^
SRV_MCAv_ (%)	supine	48.1 (15.1)^*^	35.6 (21.3)^$^	43.8 (24.0)^$^
	sitting	41.1 (21.6)^#^	9.4 (31.2)	10.9 (29.0)
	standing	47.0 (12.3)^#^	16.8 (24.5)	19.7 (27.9)

Values are mean (SD). T1-T3: time intervals from step responses corresponding to 0–5 s, 5–10 s, and 10–15 s, respectively

*SRV*_*PCAv*_ step response of the posterior cerebral artery velocity subcomponent, *SRV*_*MCAv*_ step response of the middle cerebral artery velocity subcomponent

Post-hoc ^#^*p* < 0.05 compared to T2 and T3 for same posture; ^*^p < 0.005 compared to T2 for same posture; ^$^*p* < 0.001 compared to sitting and standing for corresponding time interval

**Table 4:** Mean step responses for PCA-RAP and MCA-RAP subcomponents for time intervals T1-T3

Artery	Posture	T1	T2	T3
PCA-SRV_RAP_ (%)	supine	−62.3.(38.2)^#,$^	−105.1 (45.5)^$^	−109.2 (50.2)^$^
	sitting	−50.2 (27.8)^#^	−92.6 (39.6)	−97.7 (44.6)
	standing	−38.7 (16.4)^#^	−65.1 (26.6)	−65.8 (26.4)
MCA-SRV_RAP_ (%)	supine	−56.9 (30.6)^#,&^	−101.8 (53.7)^*^	−107.5 (71.3)^*^
	sitting	−47.0 (21.6)^#^	−87.8 (36.0)	−91.9 (37.5)
	standing	−38.3 (17.1)^#^	−72.8 (35.5)	−76.4 (39.8)

Values are mean (SD). T1-T3: time intervals from step responses corresponding to 0–5 s, 5–10 s, and 10–15 s, respectively

*PCA-SRV*_*RAP*_ step response of the resistance-area product from the posterior cerebral artery subcomponent, *MCA-SRV*_*RAP*_, step response of the resistance-area product from the middle cerebral artery subcomponent

Post-hoc ^#^*p* < 0.001 compared to T2 and T3 for same posture; ^$^*p* < 0.001 for all three postures for the same time interval; ^&^*p* < 0.05 compared to standing for the same time interval; ^*^p < 0.001 compared to standing for the same time interval

Differences due to posture can also be visualised in the PCA-SRV_CrCP_ (Fig. 2e) and MCA-SRV_CrCP_ (Fig. 2f), with post-hoc analysis showing that standing was different from supine for all three time intervals (*p* < 0.001, Table 5). For T2 and T3, MCA-SRV_CrCP_, presented differences between all three postures and PCA-SRV_CrCP_ also showed differences between sitting and standing (*p* < 0.001, Table 5).

**Table 5:** Mean step responses for PCA-CrCP and MCA-CrCP subcomponents for time intervals T1-T3

Artery	Posture	T1	T2	T3
PCA-SRV_CrCP_ (%)	supine	14.7 (22.7)^*,#^	36.8 (31.1)^*,§,#^	42.7 (36.3)^*,#^
	sitting	5.3 (14.9)	26.3 (24.9)	33.7 (31.0)
	standing	-1.2 (13.2)	6.2 (22.7)	10.4 (25.9)
MCA-SRV_CrCP_ (%)	supine	10.1 (13.3)^*,#^	29.8 (23.3)^*,#^	36.2 (28.7)^*,#^
	sitting	3.2 (9.5)	20.1 (20.0)^&^	26.2 (24.6)^&^
	standing	-4.8 (13.3)	5.2 (21.3)	10.4 (24.0)

Values are mean (SD). T1-T3: time intervals from step responses corresponding to 0–5 s, 5–10 s, and 10–15 s, respectively

*PCA-SRV*_*CrCP*_ step response of critical closing pressure from the posterior cerebral artery subcomponent, *MCA-SRV*_*CrCP*_ step response of critical closing pressure from the middle cerebral artery subcomponent

Post-hoc ^#^*p* < 0.001 compared to T2 and T3 for the same posture; ^$^*p* < 0.001 for all three postures for the same time interval; ^*^p < 0.001 compared to standing for the same time interval; ^§^*p* < 0.05 compared to sitting for the same time interval; ^&^*p* < 0.05 compared to supine and standing for the same time interval

### Step responses – effect of artery

**Fig. 3:**
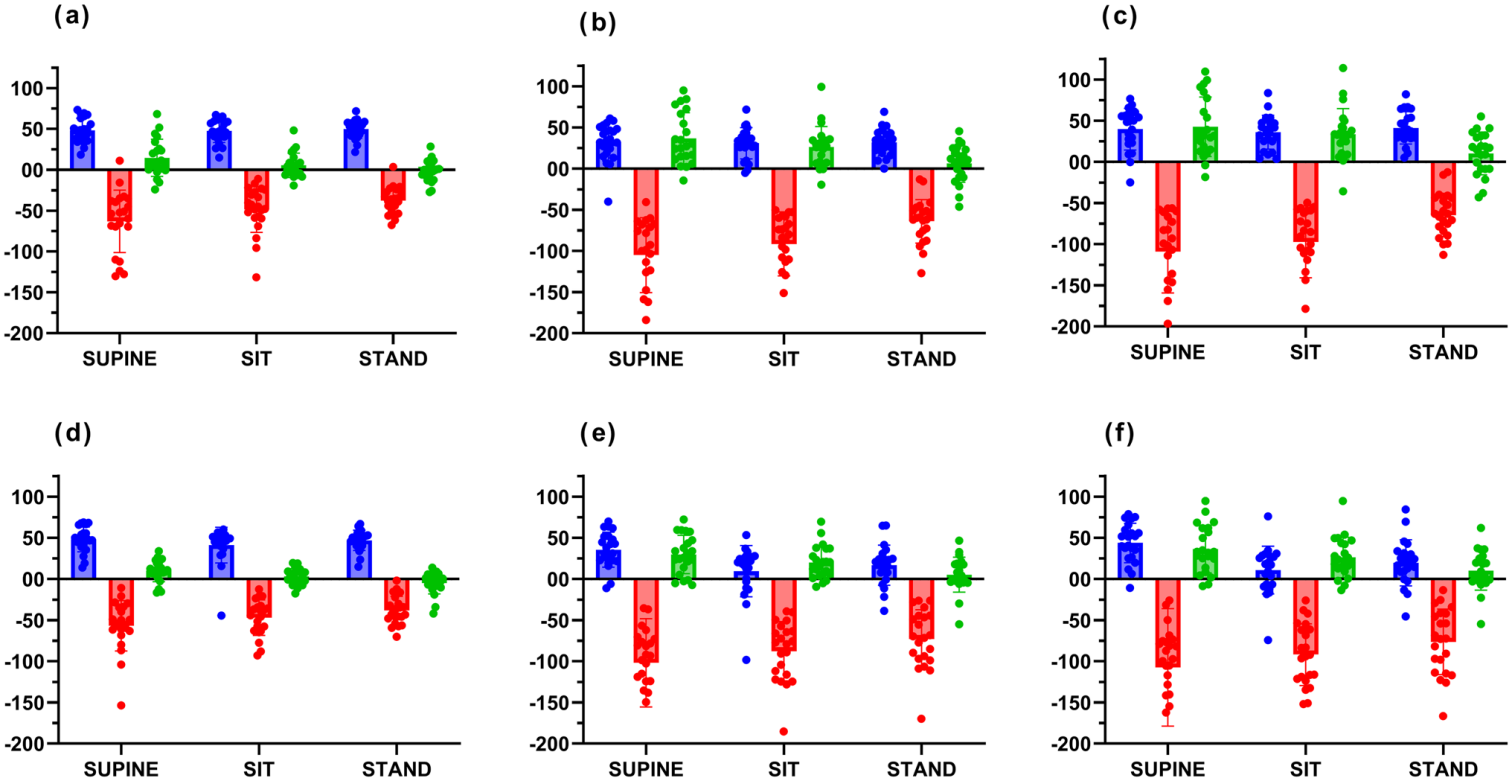
Original data points and mean values (bar amplitude) for step responses for the **a, b, c** PCA and **d,e,f** MCA at (**a, d**) T1, (**b, e**) T2 and (**c, f**) T3 for CBv (blue), RAP (red) and CrCP (green)

Main effects for differences between step responses derived from the PCA and MCA were only obtained for the SRV_MCAv_ and SRV_PCAv_, but significant interactions were observed for all three step responses (Table 2). Most of these interactions have been mentioned above. In summary, the SRV_MCAv_ (Fig. 2b, Table 3) showed greater sensitivity to express the influence of posture compared to SRV_PCAv_ (Fig. 2a, Table 3), while this was reversed for the SRV_RAP_ responses. Whilst the PCA-SRV_RAP_ showed significant differences for all three time intervals (Fig. 2c, Table 4), the MCA-SRV_RAP_ only showed differences between supine and standing, also for the three time intervals (Fig. 2d, Table 4). For SRV_CrCP_, there was not an interaction effect involving the artery distinction (Table 2), confirmed by post-hoc analysis (Table 5). SRV_CrCP_ derived from both arteries showed highly significant differences between supine and standing (*p* < 0.001, Table 5, Fig. 2e and f) for all three time intervals. For T2 and T3, marked differences were obtained for both arteries between sitting and standing (*p* < 0.001, Table 5, (Fig. 3), but less robust differences were also obtained between supine and sitting (*p* < 0.05, Table 5, (Fig. 3).

## Discussion

### Main findings

This study combined TFA and subcomponent analysis to model CBF regulation during changes in posture, also taking into consideration regional differences in CBF, represented by MCAv or PCAv, obtained with TCD. The dynamic response of MCAv/PCAv to MAP changes was decomposed into the step responses of RAP and CrCP, which can extend the information provided by the more classical step responses of MAP-MCAv and MAP-PCAv.

Our main findings were that although CBv step responses changes with posture were only significant for the MCA, RAP and CrCP step responses did change significantly with posture on both PCA and MCA territories.

### Physiological interpretation

The main hypothesis of our study was confirmed by the greater sensitivity shown by SRV_RAP_ and SRV_CrCP_ to reflect differences in posture, when compared to the more classical SRV_CBv_, for both the MCA and PCA (Fig. 2, Tables 3–5). Of considerable relevance though, is how this additional information can help to advance our understanding of the effects of posture on cerebral haemodynamics.

The performance of SRV_CBv_ for the MCA and PCA was different (Fig. 2), and it can be clearly observed that the SRV_MCAv_ showed greater differences between different postures than the PCA, as confirmed by the ANOVA (Table 3). This finding could be the result of structural differences between the two arteries. As the main branch of the anterior circulation (internal carotid artery system), the MCA has a thicker smooth muscle layer in its vascular wall and a stronger myogenic regulatory ability. This structural feature enables it to more effectively regulate resistance through contraction or relaxation in response to changes in blood pressure and maintain stable microvascular pressure. The PCA, on the other hand, belongs to the posterior circulation (basilar artery system), and its vascular wall smooth muscle layer is relatively thin, with a weaker myogenic response. The anatomical structure of the posterior circulation arteries may limit their adaptability to hemodynamic changes (Faraci and Heistad 1990; Hamner and Tan 2014; Willie et al. 2014). This may also be due to the fact that the MCA region receives a denser sympathetic innervation (Hamner and Tan 2014; Hamner et al. 2012), and the MCA supplies high metabolic areas such as the frontal lobe and parietal lobe, and local metabolites (such as CO_2_) have a stronger regulatory effect on vascular wall tension. The blood flow changes in the PCA are more affected by local metabolic factors and endothelial function (Faraci and Heistad 1990; Hamner and Tan 2014; Willie et al. 2014), and by supplying areas such as the occipital lobe with lower metabolic demands, it might have had less evolutionary pressure on its regulatory mechanism (Faraci and Heistad 1990). These theories may explain why it is more widely believed that the PCA is less able to adapt to challenges that reduce CA function, such as postural changes, than the MCA (Faraci and Heistad 1990; Hamner and Tan 2014). In agreement with these physiological considerations, a stronger dCA response to changes in posture was observed in the SRV_MCAv_ than in the SRV_PCAv_ (Fig. 2a and b). What is novel, and very intriguing though, is that PCA-SRV_RAP_ and PCA-SRV_CrCP_ showed greater sensitivity to changes in posture, compared to the MCA (Fig. 2, Tables 3–5). This finding reinforces the potential of SRV_RAP_ and SRV_CrCP_ to reflect regulatory adaptations of the cerebral circulation in health and disease. Moreover, it might also signal towards changes in the myogenic-metabolic pathways in the PCA region, compared to the MCA.

The temporal pattern of SRV_RAP_ (Fig. 2c and d) is likely to reflect the myogenic response characteristics of intracranial blood vessels to a sudden increase in MAP (Panerai et al. 2021, 2020, 2005). When MAP increases sharply, the permeability of vascular smooth muscle cell membranes to potassium and calcium ions changes, inducing the influx of Ca^2+^ and vascular smooth muscle contraction, which ultimately leads to vasoconstriction and local blood flow decrease (Faraci et al. 1989; Knot and Nelson 1998). From the SRV_RAP_ responses (Fig. 2c and d), we could speculate that moving from supine to sitting and then to standing, leads to reduced vasoconstriction due to myogenic pathways, which could be ascribed to correspondingly greater levels of sympathetic nerve activity, coupled to the reduced PaCO_2_ of the sitting and standing postures, compared to supine (Table 1) (Carey et al. 2001; Hamner and Tan 2014; Hoiland et al. 2011; Panerai et al. 2024; Willie et al. 2014). Despite the reductions in mean PCAv and MCAv from supine to sitting and standing (Table 1) though, the lack of differences in mean PCA-RAP and MCA-RAP (Table 1) do not suggest that baseline values of RAP were altered due to posture. Therefore, the underlying physiological determinants of why RAP reduces its contribution to explain the SRV_CBv_ with changes in posture, from supine to sitting and then to standing, is intriguing, and deserve more detailed investigation.

On the other hand, despite the complexity of the determinants of CrCP (Burton 1951; Panerai 2003; Panerai et al. 2024, 2021), interpretation of the changes in the temporal pattern of SRV_CrCP_ with posture is more straightforward. Moving from supine to the sitting position reduces ICP and, correspondingly, transmural pressure, which explains the reduction in mean CrCP (Table 1) (Brasil et al. 2023; Burton 1951). CrCP is also highly sensitive to PaCO_2_ (Panerai 2003), which was likely to be reduced with standing, based on the EtCO_2_ values in Table 1. These influences on baseline CrCP possibly balanced each other out and were not significantly manifested in the early phase (T1) of the SRV_CrCP_ (Fig. 2e and f, Table 5). However, very significant differences were obtained during the intermediate and final stages of the step responses (Table 5). During the T2 and T3 time intervals, the slow rise of the SRV_CrCP_ has been speculated to reflect the phenomenon of wall shear stress (Hoiland et al. 2022; Panerai et al. 2021, 2020, 2005). Our interpretation of the higher values of SRV_CrCP_ at T2 and T3 for the supine position, compared to sitting and standing (Fig. 2e and f) then, is that these are reflecting higher levels of wall shear stress, due to the higher corresponding mean velocities observed in these postures. If much needed further work confirms that the late phase of SRV_CrCP_ is expressing differences in wall shear stress, given its association with endothelial function and the release of nitric oxide (Hoiland et al. 2022), what is being demonstrated then, is the very exciting prospect that the combination of TFA with subcomponent analysis is reflecting differences in nitric oxide dynamics with changes in posture.

A better understanding of the interaction between posture and dCA will allow for further research into cerebral hemodynamics in healthy and patient populations. Older people are at risk of falls, in part due to autonomic dysfunction and decreased baroreceptor sensitivity (Alagiakrishnan 2019). In many cases, the posterior circulation is affected, such as in vertebrobasilar insufficiency (Hoiland et al. 2011), highlighting the importance of more research on the PCA involvement in these conditions. Similar considerations apply to other patient populations where the pathophysiological basis of posture-related symptoms remains limited, such as Parkinson's disease, a condition that can be associated with impaired dCA (Barnes et al. 2022).

### Methodological considerations and limitations of the study

The parameters CrCP and RAP are indirectly estimated from the instantaneous pressure–velocity relationship during each cardiac cycle, instead of being directly measured. Therefore, they are susceptible to interference from the noise and baseline drift of MAP and CBv signals, which may lead to the generation of non-physiological outliers (such as very negative CrCP). Although multi-cycle averaging can partially reduce variability, achieving stable estimates of the SRV_CrCP_ and SRV_RAP_ based on a single recording is still difficult (Panerai 2008; Panerai et al. 2024; Zhang et al. 1998). Due to the inherent challenges of determining the CrCP value for each cardiac cycle mentioned above, this can lead to a lack of consistent temporal patterns and widely dispersed estimates (Panerai et al. 2024, 2011). If not removed, these outliers may distort results and their interpretation. Previous studies usually eliminated estimates that deviate from the population mean through visual inspection. Despite the subjectivity of this procedure, it usually led to more consistent results (Panerai et al. 2021, 2020).

However, in clinical research scenarios, this manual screening method has the risk of introducing selection bias. It is worth noting that the reliability of TFA requires that the coherence function achieve statistical significance. The reduced variability of the input signal at rest (such as MAP) itself will significantly affect the accuracy of parameter estimation (Elting et al. 2020). In addition, the quality of SRV_MAP_* (Eq. 2) reconstruction is also an important influencing factor (Panerai et al. 2024). While SRV_CrCP_ may reflect the slow response process of CBF metabolic regulation (Beishon et al. 2018; Panerai et al. 2021, 2020, 2005), the differences between postures may be masked by a reduced sample size and/or insufficient recording duration.

This study recruited 22 participants, a sample size that should allow detection of changes in dCA (Brodie et al. 2009), but was not sufficient to study the effects of gender and age. Further studies with appropriate designs are needed to explore these potential influencing factors. Considering the potential application of our findings in clinical research, it would be meaningful to conduct studies comparing young and older populations. One of the participants consumed caffeine 1 h before participation, which violated the experimental protocol. This may have biased the results, but their data were not noticeably abnormal. For this reason, and in the interest of reporting population results for the largest possible sample, we have kept this individual in the analysis, but further studies of the effects of caffeine on the CrCP and RAP step responses would be of considerable interest.

Our analysis mainly focused on the effect of posture on CA and did not take CO_2_ into consideration. EtCO_2_ was lowest in the upright position (Table 2). Many studies have shown that dCA function is enhanced when EtCO_2_ is reduced (Aaslid et al. 1989; Clough et al. 2024; Davies et al. 2024; Hoiland et al. 2011; Minhas et al. 2018; Panerai et al. 2020). This may partially explain the MCAv step responses in Fig. 2, showing that the level of CBF recovery in the upright position is between those in supine and sitting, with the reduced EtCO_2_ in the standing position enhancing the dCA of MCAv. In future studies, it would be important to include EtCO_2_ as a potential co-factor in multivariate analyses to test the hypothesis that when the contribution of CO_2_ is taken into account, the deterioration of dCA in the standing position is even worse than what we have observed.

In our experiments, data were collected during spontaneous fluctuations in blood pressure during rest, without inducing a sudden change in MAP. We assumed that the MAP fluctuations were sufficient to trigger a dynamic CA response. However, the magnitude of MAP changes observed due to spontaneous physiological variability is usually relatively low (Elting et al. 2020). Therefore, the reliability of this method has been questioned compared to other methods, such as the rapid release of inflated thigh cuffs or repeated squats (Aaslid et al. 1989; Claassen et al. 2009; Simpson & Claassen 2017). Although the coherence criterion can assist us in confirming the reliability of estimates, a higher accuracy may be achieved by excluding records with minimal blood pressure changes (Elting et al. 2020) or using methods that induce larger MAP changes (Simpson and Claassen 2017). On the other hand, spontaneous MAP variability has minimal interference with underlying physiological processes and can be adopted in most clinical scenarios (Aaslid et al. 1989; Claassen et al. 2016; Zhang et al. 1998). This also makes our results have broader clinical application prospects.

Other limitations include the assumption that the cross-sectional areas of the PCA and MCA remained constant during the recording period to maintain a stable relationship between changes in CBv and corresponding changes in CBF (Serrador et al. 2000). This assumption has not been greatly questioned given the lack of large changes in MAP or EtCO_2_. On the other hand, it is possible that the MCA and/or PCA cross-sectional areas might have changed due to the gravitational effects of posture changes, with corresponding adjustments in ICP. To our knowledge, the influence of posture change on MCA and/or PCA diameters has not been reported. However, for the internal carotid artery, Xiang and colleagues performed 3-dimensional vascular ultrasound measurements of diameter values without observing significant differences between the supine and sitting positions (Xiang et al. 2023).

## Conclusion

In this study of the effects of postural changes on dCA, we evaluated the individual contributions of dCA sub-components, namely CrCP and RAP, using TFA and subcomponent analysis. It confirmed the initial hypotheses, that both the PCA and MCA mobilize different regulatory pathways (represented by CrCP and RAP) to stabilize CBv following postural changes. Due to the differences in anatomical and physiological functions between PCA and MCA, the study demonstrated differences in dCA between these vessels. These findings may provide insights for future studies to further investigate the effects of posture and CO_2_ in pathological conditions.

## Data Availability

Data available upon reasonable request to corresponding author.

## List of abbreviations

ABP: Arterial blood pressure
ANOVA: Analysis of variance
BMI: Body mass index
CA: Cerebral autoregulation
CBF: Cerebral blood flow
CBv: Cerebral blood velocity
CrCP: Critical closing pressure
CVR: Cerebrovascular resistance
dCA: Dynamic cerebral autoregulation
EtCO_2_: End-tidal carbon dioxide
ICP: Intracranial pressure
MAP: Mean arterial blood pressure
MCA: Middle cerebral artery
NMSE: Normalised mean squared error
PCA: Posterior cerebral artery
RAP: Resistance area product
SRV_CBv_: CBv step response
SRV_CrCP_: CrCP step response
SRV_MCAv_: CBv step response of MCA
SRV_PCAv_: CBv step response of PCA
SRV_RAP_: RAP step response
TCD: Transcranial Doppler ultrasound
TFA: Transfer function analysis
